# Hospital Meetings

**Published:** 1903-04-04

**Authors:** 


### HOSPITAL MEETINGS.

### HOSPITAL AND HOME FOR INCURABLE CHILDREN.

The subscribers and supporters of this hospital were not at all deterred by the stormy weather on March 28th from attending the annual meeting, and the room was very well filled when the Rev. Canon Duckworth took the chair.

The Chairman made the gratifying announcement that her Majesty the Queen had made a donation of 10 guineas towards the special building fund, and said he was sure this kind gift would prove very useful in attracting other contributions. This was not the first occasion on which her Majesty had shown her interest in the hospital, for on a previous occasion she had sent the children some very charming Christmas presents.

The report for 1902 was then read, from which it appeared that the ordinary expenditure had again exceeded the income by over £200, and had it not been for an opportune legacy the hospital would have been in an even worse plight than at present. The receipts for the past year had been rather larger, but an increase in annual subscriptions was much needed. Arrangements in connection with the lease had now been carried through, and the institution had been converted into a company, which had enabled the committee to take the new lease in the name of the company. The committee appointed to appeal for funds for the rebuilding of the hospital had collected a sum of over £2,000, not including a special donation of £500. They had succeeded in obtaining the earnest recommendation of H.R.H. the Duke of Connaught and of H.R.H. Princess Christian for their appeal and much hoped that they would be able to secure the necessary funds.

The committee alluded with much regret to the approaching departure of Mrs. Bruce, the matron, who had been obliged for the sake of her health to seek rest from her duties. The conscientious manner in which Mrs. Bruce had always fulfilled her duties and the great success which had attended her efforts was recognised by the committee with much gratitude. The chairman took occasion also to bear testimony to the exceptional qualities of the matron, and said the committee would find it hard to appoint a successor who would rival Mrs. Bruce in devotion to work and in kindness and sympathy to the inmates of the hospital.

The usual routine work of the election of officials and passing votes of thanks having been concluded, the chairman made a further appeal on behalf of the rebuilding fund which was becoming every day a matter of more immediate necessity is the present building was really almost unfit for habitation.

A pathetic little incident occurred at the close of the meeting, when half-a-dozen of the children appeared and sang the National Anthem.

